# Circulating microRNA: a novel potential biomarker for early diagnosis of Intracranial Aneurysm Rupture a case control study

**DOI:** 10.1186/1479-5876-11-296

**Published:** 2013-11-27

**Authors:** Hengwei Jin, Conghui Li, Huijian Ge, Yuhua Jiang, Youxiang Li

**Affiliations:** 1 Department of interventional neuroradiology Beijing Neurosurgical Institute and Beijing Tiantan Hospital, Capital Medical University, 6, Tiantan Xili, Chongwen, Beijing, 100050, P.R. China; 2 Department of neurosurgery, The First Hospital of Shijiazhuang City, 36, Fanxi Road, Changan District, Shijiazhuang city, P.R. China

**Keywords:** Intracranial aneurysm, Serum miRNA, Microarray analysis

## Abstract

**Objective:**

To investigate warning effect of serum miRNA for intracranial aneurysm rupture through microarray hybridization.

**Methods:**

24 were selected from 560 patients in our department and divided into group A, B, C and D. They are aneurysms with daughter aneurysms group, aneurysm without daughter aneurysms group, ruptured aneurysms group and angiography negative group. Then a microarray study was carried out using serum miRNA. Differentially expressed miRNAs were identified. Cluster analysis was performed in order to make the results looks more intuitive and potential gene targets were retrieved from miRNA target prediction databases.

**Results:**

Microarray study identified 86 miRNAs with significantly different (p < 0.05) expression levels between three experimental groups and control group. Among them 69 are up-regulated and 17 are down-regulated. All miRNAs in group A are up-regulated, while there are up and down-regulated in group B and C. A total of 8291 predicted target genes are related to these miRNAs. Bioinformatic analysis revealed that several target genes are involved in apoptosis and activation of cells associated with function of vascular wall.

**Conclusion:**

Our gene level approach reveals several different serum miRNAs between normal people and aneurysm patients, as well as among different phases of aneurysm, suggesting that miRNA may participate in the regulation of the occurrence and development of intracranial aneurysm, and also have warning effect for intracranial aneurysm rupture. All differently expressed miRNA in group A are up-regulated, which may suggesting protective function of miRNA for intracranial vascular wall.

## Background

Intracranial aneurysm is one of the leading causes of subarachnoid hemorrhage; however the pathogenesis of aneurysm is not clear. Studies show that hemodynamic, gene, infection, ageing and congenital factors may relate to its occurrence and development. Diagnosis of intracranial aneurysm is mainly depends on imaging diagnostic methods, such as CT, CTA and DSA (golden standard). Just like aneurysms of other body parts, intracranial aneurysm is characterized by the apoptosis of smooth muscle cells, degradation of the extracellular matrix, potent inflammatory response, and increased oxidative stress in the aortic wall. Infiltration by inflammatory cells may act as mediators which lead to apoptosis of vascular smooth muscle cells [1]. However, molecular mechanism of intracranial aneurysm is still unknown.

MiRNA is a novel class of small, non-coding, single-stranded RNA that negatively regulates gene expression via translational inhibition or mRNA degradation followed by protein synthesis repression. MiRNA regulates approximately 30% of the encoding genes of the human genome at the posttranscriptional level by incorporating into the RNA induced silencing complex (RISC) and preferentially binding to the 3’ untranslated region (3’UTR) of target mRNA. RISC then inhibits gene expression either by mRNA degradation or inhibiting translation [2]. Among thousands of target genes that are regulated by miRNA [3], many belong to biological pathways including immune response and apoptosis [4]. Tissue-specific and phase-specific expression is an important characteristic of miRNA expression [5]. Such different expression levels in different tissues suggest that the physiological functions of miRNA in different tissues may be different [6]. miRNA can be detected in serum in a remarkably stable form [7] and can withstand repetitive freezing and thawing cycles [7,8], making them attractive biomarkers for human diseases.

Daughter aneurysms, also known as daughter blebs, are focal bulges that are commonly found on the surface of original aneurysm walls [9]. It is considered high risk factor for the aneurysm rupture. Crompton performed a pathological study and found that 57% of ruptured intracranial aneurysms had daughter ‘bubbles’ , whereas the bubbles were only found in 16% of un-ruptured aneurysms [10]. In order to study the relationship between serum miRNA and the occurrence and development of intracranial aneurysm, we initially analyzed circulating miRNA expression differences of the four characterized groups through microarray hybridization technology. This undoubtedly laid a solid foundation for further research of warning effect of circulating miRNA for intracranial aneurysm‘s occurrence, development and rupture.

## Method

### Sample acquisition

**Figure 1:**
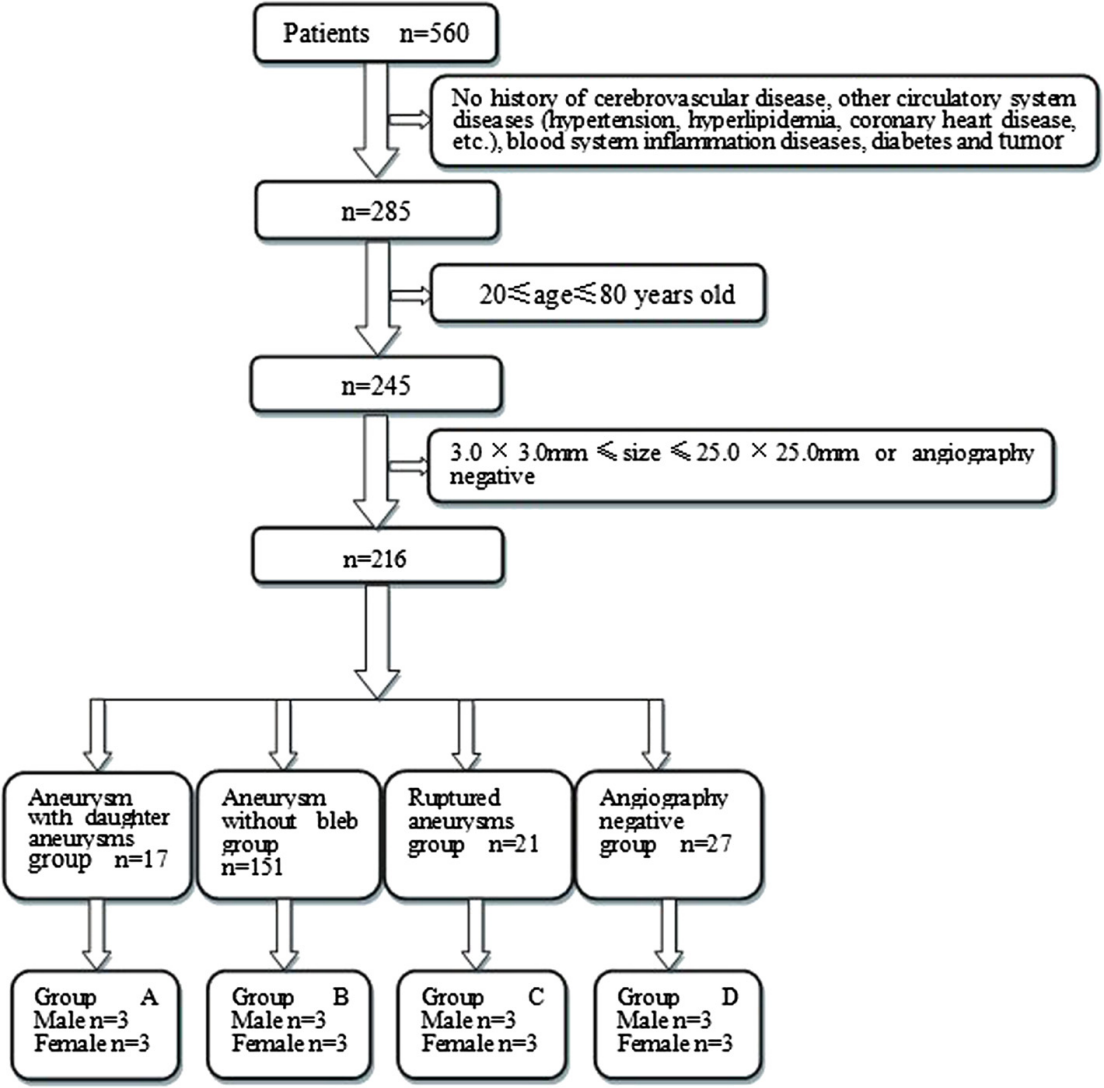
**Diagram of sample acquisition.** As revealed in this figure, 216 patients are selected from a total number of 560 under series of criterions. And then we divided them into 4 groups according to state of aneurysms. They are Aneurysm with daughter aneurysms group, aneurysm without bleb group, ruptured aneurysms group and angiography negative group. 3 males and 3 females are selected randomly from each group to form group A, B, C and D respectively.

We selected 24 out of 560 patients that went to neuro-intervention department in our hospital from December 2011 to May 2012. As is illustrated in Figure 1, 216 patients are screening out based on certain criteria (history of cerebrovascular disease like stenosis, occlusion; history of other circulatory system diseases like hypertension, hyperlipidemia, coronary heart disease, etc.; history of blood system inflammation disease; history of diabetes and tumor; age of patients greater than 80 or less than 20 years old; largest diameter of aneurysm more than 25 mm or less than 3.0 mm). Besides, considering more precise indicators of daughter aneurysm, we selected 10 (male = 3 female = 7) from 17 patients with typical blebs and ignored patients with possible controversial cystic protrusions on aneurysms. Finally, in order to make our screening criteria much striker, we enrolled the above mentioned 3 males with typical blebs. Then we selected 3 from 7 females of similar age in the same group and logically selected 3 males and 3 females on the same basis respectively from other three groups. We categorized these 24 patients into 4 groups. A is aneurysms with daughter aneurysms group. B is aneurysm without bleb group. C is the group of ruptured aneurysms that could be proved by subarachnoid hemorrhage of CT and D is angiography negative (no hemorrhage and no aneurysm) group. Each group was consist of 3 males and 3 females, and their age ranged from 24 to 78 years old (Average age = 39.7). The size of aneurysms ranged from 20 × 15 mm to 2.5 × 1.5 mm (Average size = 4.5 × 3.5 mm). There were no statistical differences (p < 0.05) among 4 groups in both the age of the patients and the size of aneurysms. In addition, 24 patients all had no history of cerebrovascular disease, circulatory system diseases (hypertension, hyperlipidemia, coronary heart disease, etc.), blood system inflammation diseases, diabetes or tumor. Aneurysms of these 18 patients mainly located in ophthalmic artery (n = 7, 38.9%), basilar artery (n = 5, 27.8%), posterior communicating artery (n = 3, 16.7%) and internal carotid artery (n = 3, 16.7%). The control group patients are angiography negative (no hemorrhage and no aneurysm). Since the hospital is the largest neurosurgery center in china, most of the patients here were referred by local doctors with consideration of operating difficulty and limited skill level. This lead to distribution difference of aneurysms compared with data reported in literatures.

The protocol of this study was carried out according to the principles of the Declaration of Helsinki and approved by the Medical Ethics Committee in Beijing Tiantan Hospital. Written informed consent was obtained from all the participants before enrolment.

### MicroRNA isolation

Blood samples of 24 subjects were taken under the condition of resting and fasting state in the morning, no intravenous transfusion or drug interference. Peripheral blood samples (2 ml) were collected into EDTA-containing tubes. Whole blood was centrifuged at 1200 g for 15 min at room temperature within 30 min after blood collection, and the supernatant was transferred into microcentrifuge tubes, followed by a second centrifugation at 12000 g for 10 min at 48°C to remove cellular debris. Plasma was then aliquoted and stored at -75°C until use. Hemosiderin absence could prove that miRNA in the serum was not released from broken red blood cells. Total RNA was extracted from plasma using the mirVana TM RNA Isolation Kit (Applied Biosystems, Foster City, CA, USA) according to the manufacturer’s specifications. Quality of the RNA samples was assessed by Nano Drop 2000 spectrophotometer (Thermo technologies). Then take 100 nanogram of the miRNA and add to a constant volume of 2 μm with nuclease-free water for hybridization.

### Microarray study

Total RNAs was labeled with the microRNA Complete Labeling and Hyb Kit (Agilent, USA) and hybridized on the Human microRNA Microarray Kit (Release 16.0, Agilent), which contains 60 000 probes for 1205 human and 144 human viral microRNAs. Hybridization signals were detected with the Agilent Microarray Scanner (Agilent) and the scanned images were analyzed using Agilent Feature Extraction Software (Agilent). Gene spring 12.0 was used to shift percentile normalization and principal component analyzed (PCA) analysis. At last, cluster analysis was carried out with Cluster 3.0 and differentially expressed miRNA was screened out.

### Bioinformatic analyses

The miRNA target prediction databases TargetScan was used to predict target genes related to these significant miRNAs. Functional classification of the target genes was carried out with Gene Ontology (GO) analysis with using Web Gestalt to create a hierarchy of the GO annotations of the predicted targets.

## Results

### PCA analysis and cluster analysis results

**Figure 2:**
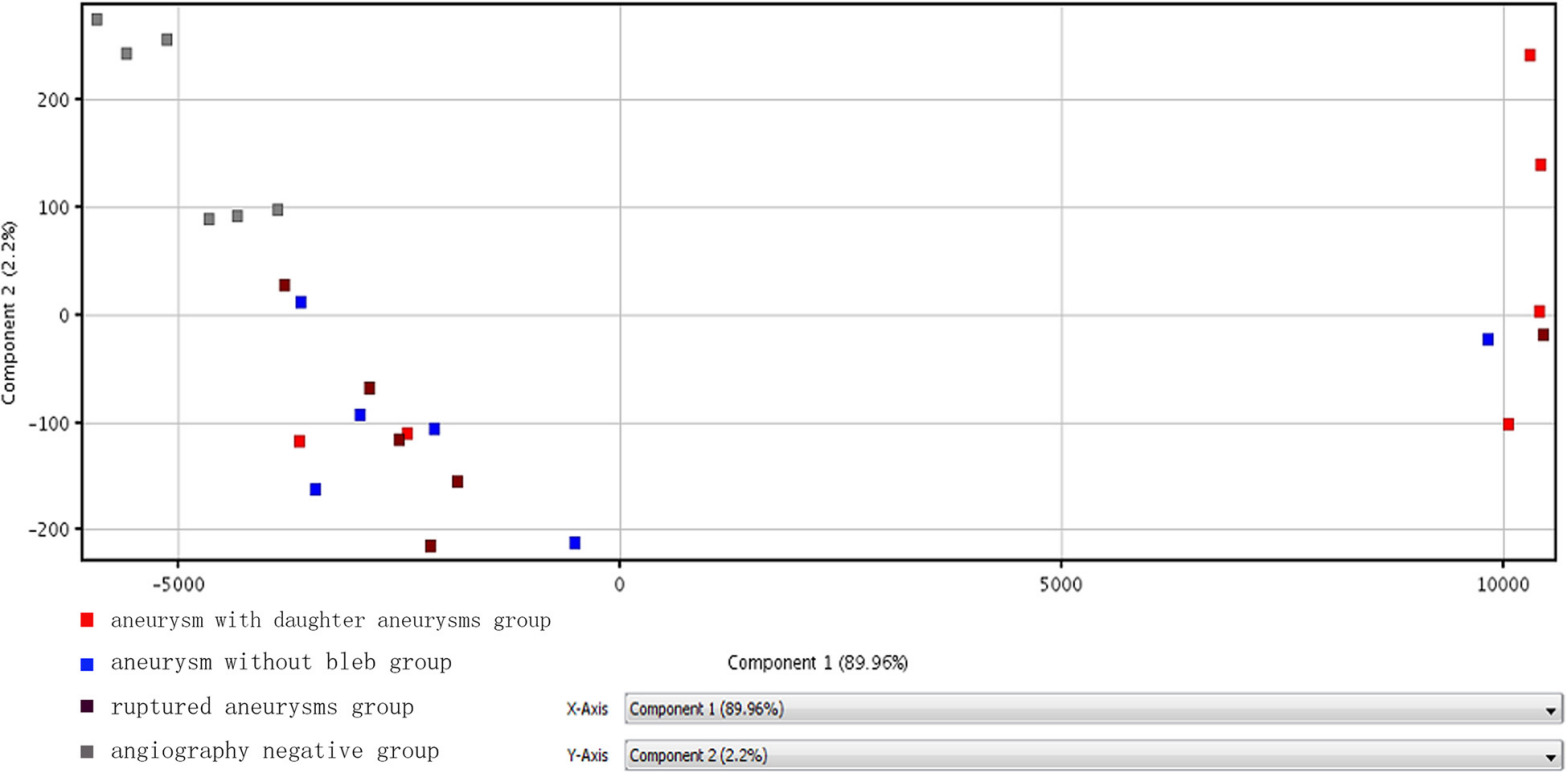
**Results of the analysis of PCA.** Red: aneurysms with daughter aneurysms group; Blue: aneurysm without bleb group; Dark: ruptured aneurysms group; Gray: angiography negative group. X-axis and Y-axis respectively stand for component 1 and 2. Small squares of the same color represent patients from the same group and have similar proportions in two components. The distribution of small squares illustrates that miRNA expression levels are different between different teams.

Gene Spring 12.0 was used to have result processed standardized, as well as principal component analyzed. Figure 2 is the results of the analysis of PCA. After the removal of secondary factor that has no statistical significance (p > 0.05), major elements (components 1 and 2) were used as standards of making new parameters. As what is illustrated in Figure 2, X-axis and Y-axis respectively stand for component 1 and 2. Small squares of the same color represent patients from the same group and have similar proportions in two components. From the distribution of small squares, we could conclude that miRNA expression is different among different groups.

**Figure 3:**
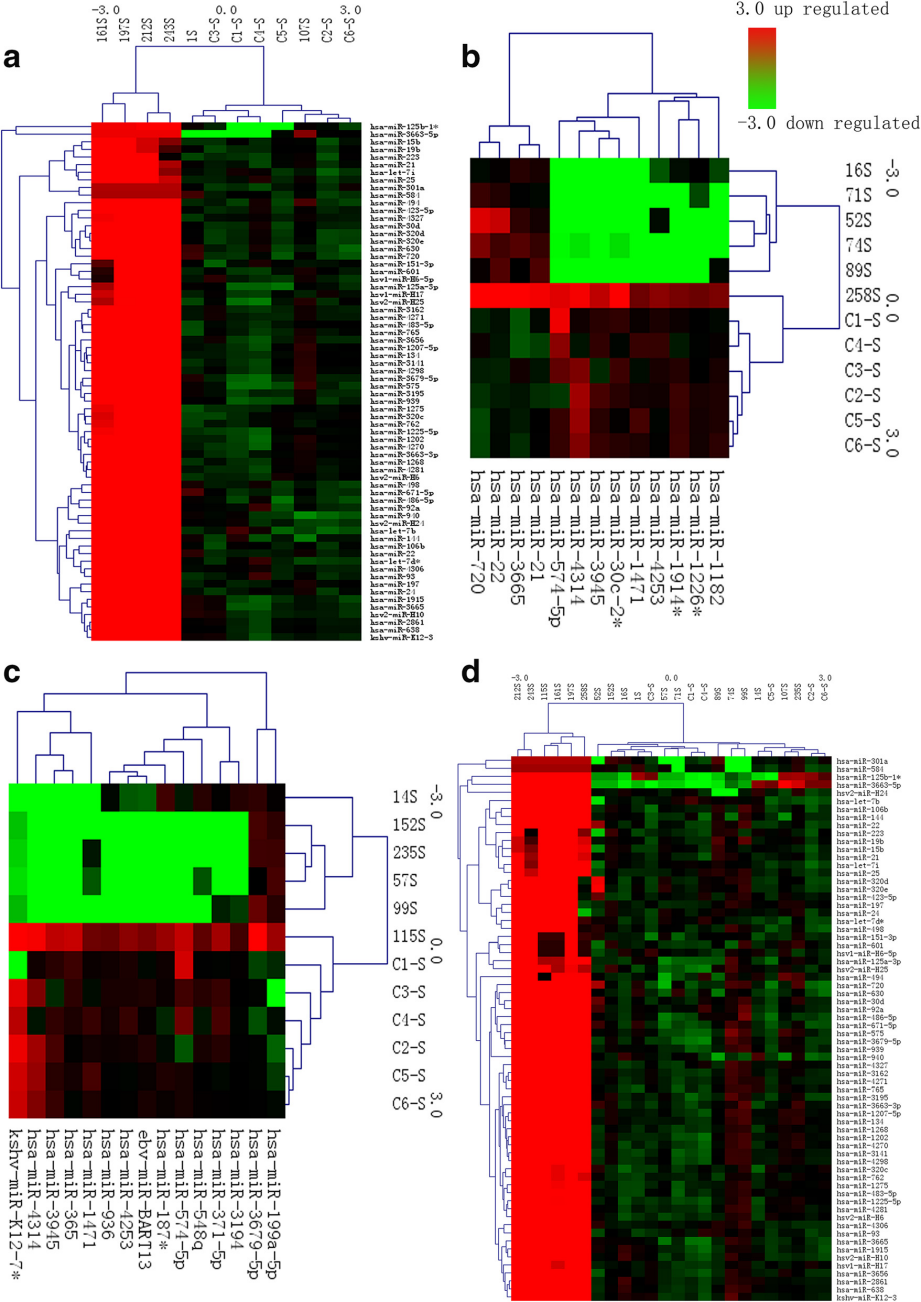
**Result of cluster analyses. (a)**, **(b)**, **(c)** and **(d)** respectively stands for differential expression results of miRNA: A vs control group, B vs control group, C vs control group and A+B+C vs control group. Red stands for up-regulated and green stands for down-regulated. C1-C6 stands for patients of control group. 197S, 212S, 243S, 161S, 1S and 107S stand for 6 patients in Group A. 16S, 52S, 258S, 74S, 71S and 89S stand for 6 patients in Group B. 115S, 152S, 235S, 57S, 99S and 14S stand for 6 patients in group C. As is revealed in the figure, miRNA expression levels are obviously different between different groups.

We have the normalized data cluster analyzed with Cluster 3.0, and some of the result is in Figure 3. We simply cited 4 differential expression result of miRNA under different selection criteria. (a), (b), (c) and (d) respectively stands for differential expression results of miRNA: A vs control group, B vs control group, C vs control group and A+B+C vs control group. Take a as an example, C1-6 in are 6 patients of control group and 197S, 212S 243S, 161S and 107S represent 6 patients of group A. Red stands for up-regulated and green stands for down-regulated. As is revealed in the figure, miRNA expression levels are obviously different among different groups.

### Differentially expressed miRNA

Screening condition is FC > =2 and p < 0.05. What’s more, at least 50% sample should be detected in the statistics that are compared. All data were processed using GraphPad Prism 5.0 (GraphPad, San Diego, CA) and SPSS software, version 17.0 (SPSS Inc, Chicago, IL).

Table 1 is the result of miRNA expression levels between intracranial aneurysms of different phases and normal people. As revealed, 86 out of all the miRNAs probes have significantly different expression level. Compared to the control group, 68 miRNA are up-regulated and none is down-regulated in team A, 4 (hsa-miR-21, hsa-miR-22, hsa-miR-3665, hsa-miR-720) are up-regulated and 9 down-regulated in team B, 2 (hsa-miR-3679-5p, hsa-miR-199a-5p) are up-regulated and 13 are down-regulated in team C (Table 1). MiRNA-21, miRNA-22 and miRNA-3665 are up-regulated in both A and B. MiRNA-3679-5p is up-regulated in both A and C. Hsa-miR-1471, hsa-miR-3945, hsa-miR-4253, hsa-miR-4314 and hsa-miR-574-5p are down-regulated in both B and C.

**Table 1:** 86 differentially expressed miRNA (p < 0.05) R: regulation of miRNA (↑ up-regulated; ↓ down-regulated and -no difference)

	***miRNA***	***A/control***			***B/control***			***C/control***		
		R	P	FC	R	P	FC	R	P	FC
1	hsa-miR-584	↑	0.000	2.2						
2	hsa-miR-301a	↑	0.001	2.2	-			-		
3	hsa-miR-3679-5p	↑	0.002	18.8	-			-		
4	hsa-miR-575	↑	0.003	18.9	-			-		
5	hsa-miR-671-5p	↑	0.003	16.1	-			-		
6	hsa-miR-22	↑	0.003	11.8	-			-		
7	hsa-miR-720	↑	0.003	23.4	-			-		
8	hsa-miR-1915	↑	0.004	11.1	-			-		
9	hsa-miR-3665	↑	0.004	11.6	-			-		
10	hsa-miR-939	↑	0.004	14.7	-			-		
11	hsa-miR-3915	↑	0.005	14.4	-			-		
12	hsa-miR-630	↑	0.005	14.8	-			-		
13	hsa-miR-320d	↑	0.005	14.4	-			-		
14	hsv2-miR-H10	↑	0.005	11.3	-			-		
15	hsa-miR-3656	↑	0.005	11.8	-			-		
16	hsa-miR-320e	↑	0.005	14.3	-			-		
17	hsa-miR-1207-5p	↑	0.005	12.8	-			-		
18	hsa-miR-125b-1*	↑	0.005	44.9	-			-		
19	hsa-miR-486-5p	↑	0.005	16.8				-		
20	hsa-miR-498	↑	0.005	14.7				-		
21	kshv-miR-K12-3	↑	0.006	10.6	-			-		
22	hsa-miR-4270	↑	0.006	12.0	-			-		
23	hsa-miR-30d	↑	0.007	14.4	-			-		
24	hsa-miR-24	↑	0.006	7.93	-			-		
25	hsa-miR-1202	↑	0.007	12.1	-			-		
26	hsa-miR-4281	↑	0.007	10.9	-			-		
27	hsa-miR-638	↑	0.007	10.2	-			-		
28	hsa-miR-2861	↑	0.007	10.0	-			-		
29	hsa-miR-494	↑	0.007	24.8	-			-		
30	hsa-miR-134	↑	0.007	11.2	-			-		
31	hsa-miR-3162	↑	0.007	13.3	-			-		
32	hsa-miR-4298	↑	0.007	13.0	-			-		
33	hsa-miR-3663-3p	↑	0.008	11.3	-			-		
34	hsa-miR-92a	↑	0.008	12.4	-			-		
35	hsa-miR-4271	↑	0.008	13.2	-			-		
36	hsa-miR-106b	↑	0.008	10.6				-		
37	hsa-miR-21	↑	0.009	7.4	-			↑		
38	hsa-miR-3141	↑	0.009	11.5	-			-		
39	hsa-miR-125a-3p	↑	0.009	14.0	-			-		
40	hsa-miR-4306	↑	0.010	9.2	-			-		
41	hsa-miR-1225-5p	↑	0.010	10.2	-			-		
42	hsa-miR-423-5p	↑	0.010	11.9	-			-		
43	hsa-miR-197	↑	0.011	7.52	-			-		
44	hsa-miR-1268	↑	0.011	9.9	-			-		
45	hsv2-miR-H25	↑	0.012	7.7	-			-		
46	hsv2-miR-H24	↑	0.013	11.9	-			-		
47	hsa-miR-25	↑	0.013	6.8	-			-		
48	hsa-miR-93	↑	0.014	8.1	-			-		
49	hsa-miR-765	↑	0.014	13.5	-			-		
50	hsv2-miR-H6	↑	0.015	9.0	-			-		
51	hsa-miR-940	↑	0.016	14.0	-			-		
52	hsa-let-7d*	↑	0.016	8.4	-			-		
53	hsa-miR-19b	↑	0.017	5.1	-			-		
54	hsa-miR-483-5p	↑	0.018	10.1	-			-		
55	hsa-let-7i	↑	0.017	8.3				-		
56	hsa-miR-4327	↑	0.019	11.2	-			-		
57	hsv1-miR-H17	↑	0.019	6.8	-			-		
58	hsa-miR-1275	↑	0.021	9.5	-			-		
59	hsa-let-7b	↑	0.022	10.0	-			-		
60	hsa-miR-15b	↑	0.023	5.3	-			-		
61	hsa-miR-320c	↑	0.023	8.3	-			-		
62	hsa-miR-144	↑	0.025	8.0	-			-		
63	hsa-miR-762	↑	0.027	7.4	-			-		
64	hsa-miR-223	↑	0.030	4.4	-			-		
65	hsa-miR-3663-5p	↑	0.042	9.3	-			-		
66	hsa-miR-151-3p	↑	0.043	7.0	-			-		
67	hsa-miR-601	↑	0.045	7.2	-			-		
68	hsv1-miR-H6-5p	↑	0.045	5.8	-			-		
69	hsa-miR-1471	-			↓	0.000	25.9	-		
70	hsa-miR-3945	-			↓	0.002	14.2	-		
71	hsa-miR-574-5p	-			↓	0.004	16.5			
72	hsa-miR-4314	-			↓	0.004	14.5	-		
73	hsa-miR-1914*	-			↓	0.008	13.0	-		
74	hsa-miR-30c-2*	-			↓	0.010	8.3			
75	hsa-miR-22	-			↑	0.022	3.1			
76	hsa-miR-720	-			↑	0.027	4.3			
77	hsa-miR-1182	-			↓	0.028	6.3			
78	hsa-miR-4253	-			↓	0.029	6.7			
79	hsa-miR-3665	-			↑	0.034	2.6			
80	hsa-miR-1226*	-			↓	0.034	6.7			
81	hsa-miR-21	-			↑	0.039	2.1			
82	hsa-miR-3945	-			-			↓	0.002	14.7
83	hsa-miR-4314	-			-			↓	0.003	14.9
84	hsa-miR-365	-			-			↓	0.003	12.6
85	ebv-miR-BART13	-			-			↓	0.004	12.8
86	hsa-miR-4253	-			-			↓	0.005	11.6
87	hsa-miR-936	-			-			↓	0.008	12.5
88	hsa-miR-574-5p	-			-			↓	0.014	9.9
89	hsa-miR-1471	-			-			↓	0.019	9.0
90	hsa-miR-187*	-			-			↓	0.032	7.5
91	hsa-miR-548q	-			-			↓	0.038	7.0
92	hsa-miR-3194	-			-			↓	0.040	7.9
93	hsa-miR-371-5p	-			-			↓	0.045	5.8
94	kshv-miR-K12-7*	-			-			↓	0.046	8.7
95	hsa-miR-199a-5p	-			-			↑	0.046	3.8
96	hsa-miR-3679-5p	-			-			↑	0.048	3.3

P: Significant P value, the smaller P is, the bigger the difference will be. FC: Absolute value of difference between groups. As is revealed, 86 out of all the miRNAs probes have significantly different expression level. Compared with control group, 68 miRNA are up-regulated and none is down-regulated in team A, 4 are up-regulated and 9 down-regulated in team B, 2 are up-regulated and 13 are down-regulated in team C.

*means low expression. It is a symbol based on naming rule of miRNA.

### Bioinformatic analyses

A total number of 8291 predicted miRNA target genes were related to these miRNAs.

And the pathways between DNA, protein and miRNA are very complicated. Every miRNA is involved in the regulation of multiple pathways and the number ranges from a few to hundreds. In the same way, one pathway is regulated by several miRNAs. This indicates that each miRNA has different biological effects in different tissues. From the perspective of gene expression process, these miRNAs are involved in the process of transcription, post-transcription modification, translation and post-translation modification; From the view of biological effects, they are involved in biological molecular activation, transportation of molecular pathways, as well as cell proliferation, differentiation and apoptosis. Considering possible factors such as aneurysm formation, development and rupture, researchers selected some of the miRNAs that related to myosin generation, vascular endothelial growth and vascular smooth muscle cell apoptosis. For example, hsa-miR-22 regulates smooth muscle cell proliferation and transcription factor activity negatively. Hsa-miR-21 induces apoptosis by extracellular signals. Hsa-miR-720 is involved in actin cytoskeleton organization and biogenesis (Table 2).

**Table 2:** 9 of the target genes that may be related to the formation, development and rupture of aneurysm

***miRNA***		***Symbol***	***GO description***
N	R		
hsa-miR-22	↑	TRIB1	Negative regulation of smooth muscle cell proliferation/negative regulation of transcription factor activity
hsa-miR-671-5p	↑	SSX3	Positive regulation of smooth muscle cell proliferation/vascular endothelial growth factor receptor signaling pathway
hsa-miR-720	↑	ARFIP2	Actin cytoskeleton organization and biogenesis
hsa-miR-365	↓	BCL6B	Negative regulation of transcription from RNA polymerase II promoter
hsa-miR-498	↑	EP300	Positive regulation of transcription factor activity/positive regulation of transcription from RNA polymerase II promoter
hsa-miR-574	↓	EDN1	Positive regulation of smooth muscle cell proliferation/blood vessel morphogenesis
hsa-miR-106b	↑	CHAF1A	Vascular endothelial growth factor receptor activity/vascular endothelial growth factor receptor signaling pathway
hsa-miR-21	↑	PDCD6	Induction of apoptosis by extracellular signals
hsa-miR-936	↓	FMN2	Actin cytoskeleton organization and biogenesis/actin binding

N: Name of miRNA; R: Regulation of miRNA(↑up-regulated and ↓down-regulated) P: Significant P value, the smaller P is, the bigger the difference will be. Symbol: Name abbreviation of the regulated gene; GO description: Brief introduction of the protein translated from the gene.

## Discussions

The pathogenesis of aneurysm may be related to heredity, infection, hypertension, congenital factors, aging, etc. Functional in vitro studies have shown that miRNA is critical for endothelial cell gene expression and function. It is estimated that more than 1,000 different miRNAs exist in humans, and many are expressed in a tissue- and/or cell-specific manner [11,12]. Their expression patterns are reflections of underlying pathophysiologic processes. And interestingly, miRNA can be detected in serum or in plasma in a remarkably stable form. Circulating miRNAs can withstand repetitive freezing and thawing cycles, making them attractive as potential biomarkers for diverse human diseases [7,8]. An ideal biomarker should be reproducibly measurable with high sensitivity and specificity for the clinical outcome of interest and should reflect an important pathogenetic process. Circulating miRNAs are exciting as potential biomarkers because they fulfill many of these criteria. The origins or sources of circulating miRNA are largely unknown. Some hypotheses have proposed that they are secreted in membrane-bounded-vesicles (apoptotic bodies, microvesicles, exosomes, etc. [13]), while others stated that they are produced by-products of dead cells [14]. Most of previous study of miRNA in bioscience extracted miRNA from cell of tissue, while the author and his partners investigated briefly the relationship between serum miRNA and intracranial aneurysm initially.

We reviewed some of current knowledge about the role of miRNA in endothelial cells with emphasis on the regulation of cellular senescence, angiogenesis, and vascular inflammation. Plasma miRNA-208a seems to be an ideal biomarker of AMI [15]. Mian Wang et al. proved that microRNA-21 regulates vascular smooth muscle cell function via targeting tropomyosin in arteriosclerosis obliterans of lower extremities [16]. Hsa-miRNA-566 is related to inflammatory immune responses of circulation system [17]; MiRNA-99b to TGF-β-mediated cell extracellular matrix [18] and hsa-miR-1180 to cell apoptosis [19]. Existing researches have shown that the expression level of miRNAs (miR-133a, miR-133b, miR-146a, miR-181a*, miR-204, miR-21, miR-30c-2*, miR-331-3p) between aortic aneurysm patients and normal person are statistically different [20,21]. And their expression difference between intracranial aneurysm patients and normal people are still unknown. The formation of aneurysm can be a result of multiple causes. When internal and external factors act on artery wall, miRNA expression level changes to regulate target genes to cope with the stimulations. As a result, original balance was destroyed, protein and extracellular matrix was produced abnormally. All this caused structural changes in arterial wall and the formation of aneurysm. In return, all the artery wall changes affect the expression level of miRNA. This supposition is also the theoretical foundation of our study.

We screen out 86 differentially expressed miRNAs, among which miRNA21 regulates vascular smooth muscle cell function via targeting tropomyosin [16]. Different group represent different phase of aneurysm. 68 miRNA are up-regulated and none is down-regulated in team A; 4 are up-regulated and 9 down-regulated in team B; 2 are up-regulated and 13 are down-regulated in team C. So it is plausible to hypothesize that aneurysm initiation, growth and rupture have different molecular mechanisms and further study needs to be done to find the variation trend. MiRNA-21, miRNA-22 miRNA-720 and miRNA-3665 are up-regulated in both A and B. MiRNA-3679-5p is up-regulated in both A and C. Hsa-miR-1471, hsa-miR-3945, hsa-miR-4253, hsa-miR-4314 and hsa-miR-574-5p are down-regulated in both B and C. These miRNAs may play more important role in the regulation of aneurysm formation and development.

Aneurysm blebs have been identified as a risk factor for increased risk for future rupture [22,23]. The presence of multiple lobes or a daughter sac is more common in previously ruptured aneurysms compare to un-ruptured aneurysms [24]. Tsukahara reported a global rupture rate of 3.42% per year and a rupture rate of 28.3% per year in aneurysms that contained blebs [22]. Therefore, to some extent, we could define group A as high rupture risk group. Differentially expressed miRNAs in group A are all up-regulated, among which many participated in the activity of vascular smooth muscle, blood vascular endothelium and growth factor, etc. For example, hsa-miR-106b, hsa-miR-22 and hsa-miR-498 regulated vascular endothelial growth factor receptor activity; hsa-miR-671 positively regulates proliferation of smooth muscle cells. This may imply protective function of these miRNA for intracranial aneurysm rupture.

Over 700 miRNAs have been identified in the human genome, of which 20% to 30% regulate human protein coding genes [2]. A total number of 8291 predicted miRNA target genes were related to these 86 miRNAs. GO analysis revealed the function of these genes. In Table 2, we listed 9 of the miRNAs (p < 0.01), target genes and corresponding proteins that may be related to the formation and development of intracranial aneurysm. As revealed, hsa-miR-22 may negatively regulate smooth muscle cell proliferation through TRIB1; hsa-miR-574 has positive regulation function on smooth muscle cell proliferation and blood vessel morphogenesis through EDN1; hsa-miR-21 could induce of cell apoptosis by using extracellular signals. Hsa-miR-720, hsa-miR-365, hsa-miR-936 respectively has important regulatory mechanism. Mutual interactions among these genes and proteins may be the real causes of aneurysm occurrence, development and rupture.

Our study had several limitations. Due to small sample size and some other factors such as individual differences, there is a certain degree of bias. For example, the distribution of points in principle components analysis result reveals obvious bias. The classification of high risk rupture group (aneurysms with daughter aneurysms group) is lack of literature basis and specific criteria. In addition, result of the experiment was not validated with RT-PCR and we will next do this following work. Much effort is needed before miRNA could be used as biomarkers for the diagnosis of intracranial aneurysm rupture.

## Conclusions

This study is initial exploration of the relationship between different phases of intracranial aneurysm by using microarray hybridization. Our gene level approach reveals several different serum miRNAs between normal people and aneurysm patients, as well as among different phases of aneurysm, suggesting that miRNA may participate in the regulation of the occurrence and development of intracranial aneurysm, and also have warning effect for intracranial aneurysm rupture. Up regulation of all miRNAs in group A suggest protective function for vascular wall. While more specific functions, the relationship among the 86 miRNAs and their connection with target genes require much efforts before miRNA expression analysis was used in the clinical diagnosis and treatment of intracranial aneurysm.

## Abbreviations

RISC: RNA induced silencing complex; PCA: Principal component analyzed; GO: Gene ontology; miRNA: microRNA.

## Competing interests

Each author stated that this work contains no libelous or unlawful statements and does not infringe any other personal or proprietary right of others, nor contains any fraudulent, plagiarized or incorrectly attributed material. Each author identified no financial interests or affiliations with institutions, organizations, or companies relevant to the manuscript. No competing financial interests exist.

## Authors’ contributions

YJ conceived the study and had main responsibility for the development of protocol and flow chart, data collection, and management. CL and participated in developing the flow chart and carried out microarray study of miRNA. HG participated in developing the flow chart and was responsible for data collection. HJ reviewed the protocol, had main responsibility for data preparation, analysis, and interpretation, and wrote the first draft of the paper. YL is the guarantors and participated in its design and coordination and helped to draft the manuscript. All authors read and approved the final manuscript.
